# Capsaicin induces ferroptosis of NSCLC by regulating SLC7A11/GPX4 signaling in vitro

**DOI:** 10.1038/s41598-022-16372-3

**Published:** 2022-07-14

**Authors:** Xiao-Yan Liu, Dong-Guang Wei, Rong-Shan Li

**Affiliations:** 1 Department of Pulmonary and Critical Care Medicine, The Affiliated People’s Hospital of Shanxi Medical University, Shanxi Provincial People’s Hospital, Taiyuan, 030012 People’s Republic of China; 2 Department of Nephrology, The Affiliated People’s Hospital of Shanxi Medical University, Shanxi Provincial People’s Hospital, Taiyuan, 030012 People’s Republic of China

**Keywords:** Cancer, Molecular biology, Oncology

## Abstract

NSCLC is the first cause of cancer-related deaths in China and threatens life expectancy of the people. Novel drugs and treatment strategies are urgently required. Capsaicin is noticed as a potential new drug for lots of tumors due to its anti-proliferative effect on cancer cells. Our study evaluated the roles of capsaicin in NSCLC cells (A549 and NCI-H23) and further explored its underlying mechanisms. Effect of capsaicin treatment on cell viability was determined by MTT assay and IC50 values for A549 and NCI-H23 cells were ascertained. The iron kit detected the total iron levels and the ferric divalent ions levels in A549 and NCI-H23 cells. GSH kit was used to detect the expression of GSH in A549 and NCI-H23 cells. Additionally, mRNA and protein levels of SLC7A11 and GPX4 were analyzed by real-time PCR and western blot analysis. Through MTT assay, we found that 200 μM capsaicin in cultured A549 cells for 48 h could reach the IC50 value, and the condition was 100 μM and 48 h for NCI-H23 cells. Capsaicin increased total iron levels and ferrous ion levels in A549 and NCI-H23 cells in contrast with the control group, whereas the levels of GSH was reduced in contrast with the control group. Besides, mRNA and protein levels of SLC7A11 and GPX4 were decreased significantly in A549 and NCI-H23 cells treated with capsaicin in contrast with the control group. Our study indicated that capsaicin inhibited the proliferation of A549 and NCI-H23 cells and induced ferroptosis by inactivating SLC7A11/GPX4 signaling. Capsaicin could be used as a potential anticancer agent in the treatment of NSCLC.

## Introduction

Because of its spicy taste, chili pepper is often used as one of the condiments in Chinese food, such as hotpot. The main ingredient of chili pepper is capsaicin (trans-8-methyl-N-vanillyl-6-nonenamide, C_18_H_27_NO_3_), which is listed in the United States Pharmacopeia^1^. It was reported that capsaicin has a number of medical functions, including anti-oxidation, anti-inflammation, cardiovascular disease prevention, gastrointestinal mucosal protection, and so on^2^. Previously, lots of studies showed that capsaicin exerts anti-cancer effect in many malignant tumors in vitro, such as non-small cell lung cancer (NSCLC), liver cancer, prostate cancer and so on^3–5^. Studies in vivo also confirmed that capsaicin has anti-tumor effects^6^. Critically, capsaicin concentrates its anti-proliferative and cytotoxic effects only on malignant cancer cells without any damages to normal cells, which is different with traditional chemotherapeutic drugs. Capsaicin plays its anti-tumorigenic role by inhibiting proliferation of cancer cells, inducing cell cycle arrest, inhibiting tumor angiogenesis and promoting cancer autophagy^7–9^. Although the inhibitory effect of capsaicin on NSCLC was reported recently^10^, its specific molecular mechanism is still unclear.

Ferroptosis is a novel kind of programmed cell death induced by iron-dependent lipid peroxide accumulation^11^. Solute carrier family 7 member 11 (SLC7A11) is regarded as a vital regulator of ferroptosis. Suppression of SLC7A11 significantly inhibited the growth of tumor cells by leading to ferroptosis^12,13^, while activation of SLC7A11 correlated to tumor progression positively. It was reported that both radiotherapy and immunotherapy could induce ferroptosis of tumor cells by down-regulating SLC7A11^14^. In the downstream of SLC7A11, glutathione peroxidase 4 (GPX4) is another critical regulator of ferroptosis, which could decrease lipid peroxides^15^. It was reported that suppression of GPX4 led to the inhibition of tumor progression in vivo^16^. Nevertheless, it was found that ferroptosis is inhibited in lung cancer cells. Lai et al.^17^ reported that pulmonary tumor cells inhibit ferroptosis by up-regulating of GPX4 expression. GPX4 activation could weaken mitochondrial injuries induced by ferroptosis and promote lung cancer progression, while the knockdown of GPX4 could promote ferroptosis. Jiang et al.^18^ discovered that lung cancer cells resist ferroptosis by regulating lipid metabolism. In spite of these findings, the molecular mechanism of ferroptosis suppression in NSCLC is still unknown. Besides, many chemotherapeutic agents exert its effects for NSCLC via ferroptosis. It was found that cisplatin could induce ferroptosis of A549 by inhibiting GPX4. Cisplatin and Erastin could play synergistic role to lung cancer^19^. However, the side effects of chemotherapy drugs are obvious, since normal cells are also damaged during the anti-tumor process^20^. Hence, it is urgent to find novel drugs for NSCLC which could induce ferroptosis.

In this research, we studied the therapeutic role of capsaicin on NSCLC cells and discussed its probable molecular mechanisms. By using A549 and NCI-H23 human lung adenocarcinoma cancer cell lines, we firstly adopted 3-(4,5-dimethylthiazol-2-yl)-2,5-diphenyltetrazolium bromide (MTT) assay to assess the cytotoxic effects of capsaicin on cell viability. And then, we checked the changes of total iron, divalent iron, and glutathione (GSH) levels in A549 and NCI-H23 cells after the capsaicin treatment. Finally, we analyzed both mRNA and protein expression levels of SLC7A11 and GPX4 in A549 and NCI-H23 cells after the capsaicin treatment. The aim of this study is to provide a theory basis for the therapeutic potential of capsaicin for NSCLC patients and make clear its mechanisms.

## Materials and methods

### Cell culture and MTT assay

A549 and NCI-H23 cells were purchased from the American Type Culture Collection (ATCC, Manassas, VA, USA). We cultivated these two kinds of cells with high glucose Dulbecco’s modified Eagle’s medium (DMEM; Gibco; Thermo Fisher Scientific, Inc.) and 10% fetal bovine serum (FBS; Gibco; Thermo Fisher Scientific, Inc.) in a humidified environment of 5% CO_2_ at 37 °C.

The A549 and NCI-H23 cells (1 × 10^4^ cells/well) were respectively cultured in 96-well plates and treated with 0, 50, 100, 200, and 300 μM capsaicin (A10178, AdooQ) concentrations for 24 and 48 h. After treated with capsaicin, MTT (5 mg/mL in PBS, 10 µL, Sigma-Aldrich) was added to every well and cultured at 37 ℃ for 3 h without light. Next, the supernatant was discarded and the formed formazan crystals were dissolved in 100 μL 100% dimethyl sulfoxide (DMSO, Sigma-Aldrich). Finally, the absorbance was detected at 570 nm by using the analysis apparatus (Molecular Devices, Sunnyvale, CA, USA). The IC50 values for A549 and NCI-H23 cells treated by capsaicin were ascertained for the following studies. To differentiate apoptosis and ferroptosis, we used ferrostatin-1 (Fer-1, a suppressant of ferroptosis, Sigma-Aldrich, 1 μM) and Z-VAD-FMK (ZVF, a suppressant of apoptosis, Sigma-Aldrich, 100 μM), and observed the changes of A549 and NCI-H23 cells viability respectively. The concentrations of Fer-1 and ZVF here were decided according to the previous reports^21,22^.

After confirmed the IC50 values of A549 and NCI-H23 cells, the cells were divided into 3 groups respectively for the following researches: (1) the control group, (2) the capsaicin group, (3) the capsaicin + ferrostatin-1 group.

### Iron concentration detection

We detected total iron levels in each group of A549 and NCI-H23 cells by using the iron assay kit (MAK025, Sigma-Aldrich) in accordance with the operating manual.

### Fe^2+^ assay

We detected divalent iron levels in each group of A549 and NCI-H23 cells by applying the iron assay kit (ab83366, Abcam, UK) in accordance with the operating manual.

### GSH assay

GSH levels in each group of A549 and NCI-H23 cells were assessed by applying a GSH test kit (BioVision Inc., Milpitas, CA, USA) in accordance with the operating manual.

### Total RNA extraction and real-time PCR

As the methods published in the previous reports^23^, total RNA was isolated from each group of A549 and NCI-H23 cells respectively. The mRNA levels of SLC7A11 and GPX4 were analyzed by real-time PCR using Power SYBR™ Green PCR Master Mix (Thermo Fisher Scientific). The PCR conditions were: 1 cycle of 2 min at 50 °C and 2 min at 95 °C, 40 cycles of 15 s at 95 °C and 1 min at 60 °C. The relative gene expressions were normalized to GAPDH, and those relative to the calibrator were given by the formula 2^−ΔΔCt^. The following primer pairs were used for real-time PCR: GPX4 forward 5ʹ-AGA GAT CAA AGA GTT CGC CG-3ʹ, reverse 5ʹ-TTG TCG ATG AGG AAC TGT GG-3ʹ; SLC7A11 forward 5ʹ-GGA TTG GCT TCG TCA TCA CT-3ʹ, reverse 5ʹ-ATA ATC AAC CCG CGG TAC TC-3ʹ; GAPDH forward 5ʹ-TGT TCG TCA TGG GTG TGA AC-3ʹ, reverse 5ʹ-ATG GCA TGG ACT GTG GTC AT-3ʹ.

### Western blotting analysis

Western-blotting was implemented as the procedures reported by Fathi et al.^24^. We extracted protein from each group of A549 and NCI-H23 cells by using a RIPA protein extraction buffer (Beyotime, Shanghai, China). By using the BCA protein test kit (Beyotime, Shanghai, China), we detected protein concentrations. And then, we separated and electrotransferred equal amounts of protein (40 μg) from each sample onto polyvinylidene difluoride (PVDF) membranes. After blocked and incubated with the primary antibodies, the membranes were then incubated with secondary antibodies. In the experiment, we used the antibodies as follows: anti-SLC7A11 antibody (1:1500, ab175186, Abcam), anti-GPX4 (1:2000, ab125066, Abcam), anti-β-actin antibody (1:1000, ab8227, Abcam). Finally, detection was done by chemiluminescence (Millipore Corporation, Temecula, CA, USA).

### Statistical analysis

Statistical analysis was performed by GraphPad Prism Software (Version 8.0). Data obtained from each experiment in vitro were presented as means ± SD. Differences were identified by one-way analysis of variance (ANOVA) followed by Tukey’s post hoc test, and *P* < 0.05 was regarded as significance. All experiments were repeated thrice independently.

## Results

### Capsaicin suppressed the cell viability of A549 and NCI-H23 via apoptosis-independent and ferroptosis-dependent manner

**Figure 1:**
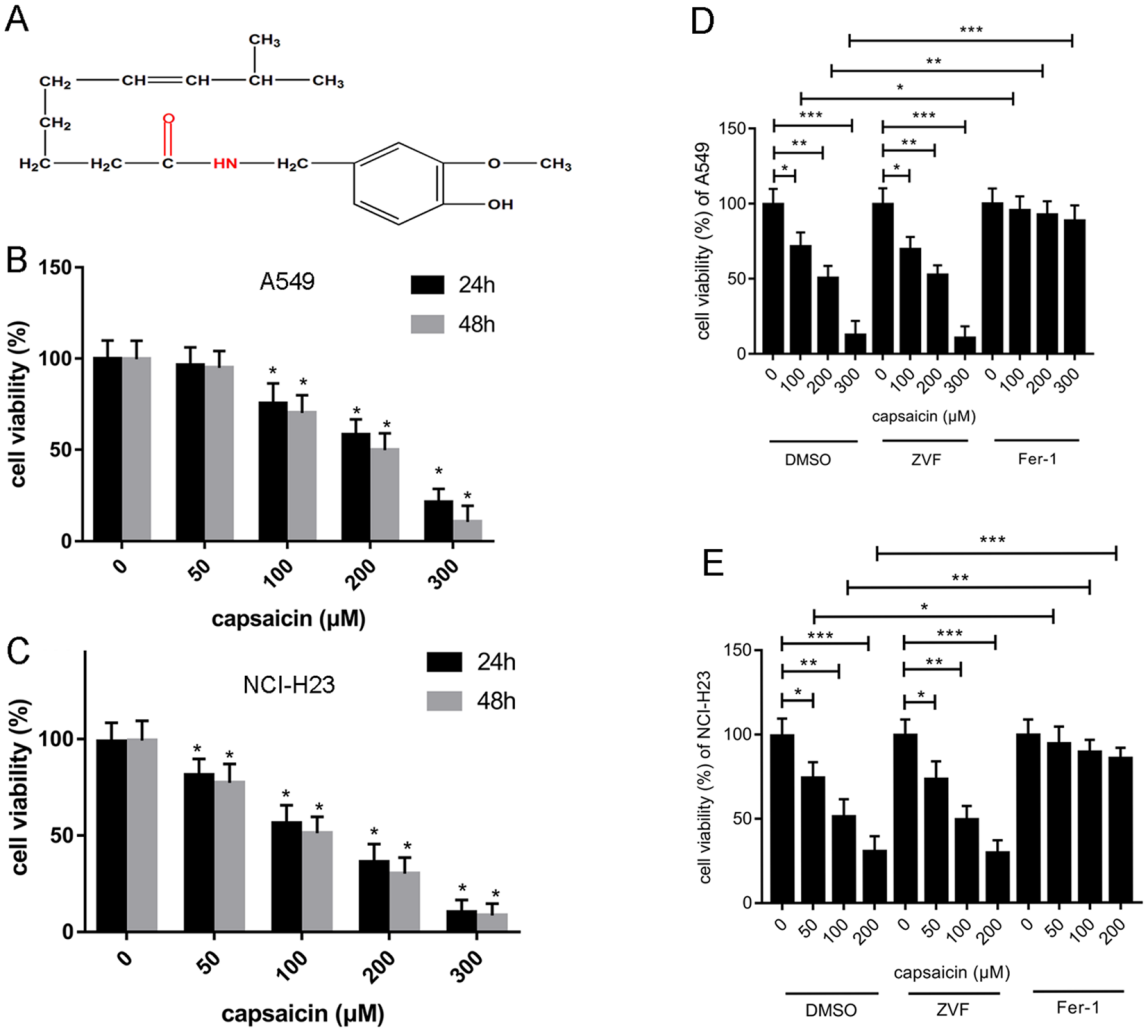
Capsaicin induced ferroptosis of A549 and NCI-H23 cells. (**A**) The chemical formula of capsaicin. (**B** and **C**) The cell viabilities of A549 and NCI-H23 cells which were treated by capsaicin (0 μM, 50 μM, 100 μM, 200 μM and 300 μM) were detected by MTT assay. (**D** and **E**) The cell viability of A549 and NCI-H23 cells which were treated with capsaicin and ZVF (Z-VAD-FMK, 100 μM)/Fer-1 (Ferrostatin-1, 1 μM) were analyzed by MTT assay. The experiments were repeated thrice. (*P < 0.05, **P < 0.01, ***P < 0.001).

The chemical formula of capsaicin was demonstrated in Fig. 1A. We investigated the effect of capsaicin on A549 and NCI-H23 cells respectively by MTT. The results demonstrated that 100 μM, 200 μM, and 300 μM capsaicin decreased the cell viability of A549 significantly both 24 h and 48 h, whereas 50 μM capsaicin did not have significant anti-proliferative effect on A549. Capsaicin (200 μM) could reduce the viability of A549 cells and reach IC50 at 48 h (Fig. 1B). Besides, 50 μM, 100 μM, 200 μM, and 300 μM capsaicin reduced the cell viability of NCI-H23 significantly both 24 h and 48 h, and NCI-H23 cells reached IC50 with 100 μM capsaicin at 48 h (Fig. 1C). After treated together with ferrostatin-1 and capsaicin for 48 h, the cell viability of A549 and NCI-H23 were almost unaffected, which were significantly different with capsaicin alone. However, after 48 h treatment, the apoptosis inhibitor Z-VAD-FMK could not alleviate the capsaicin-induced decline in cell viability of A549 and NCI-H23 (Fig. 1D,E).

### Capsaicin induces ferroptosis of A549 and NCI-H23 cells

**Figure 2:**
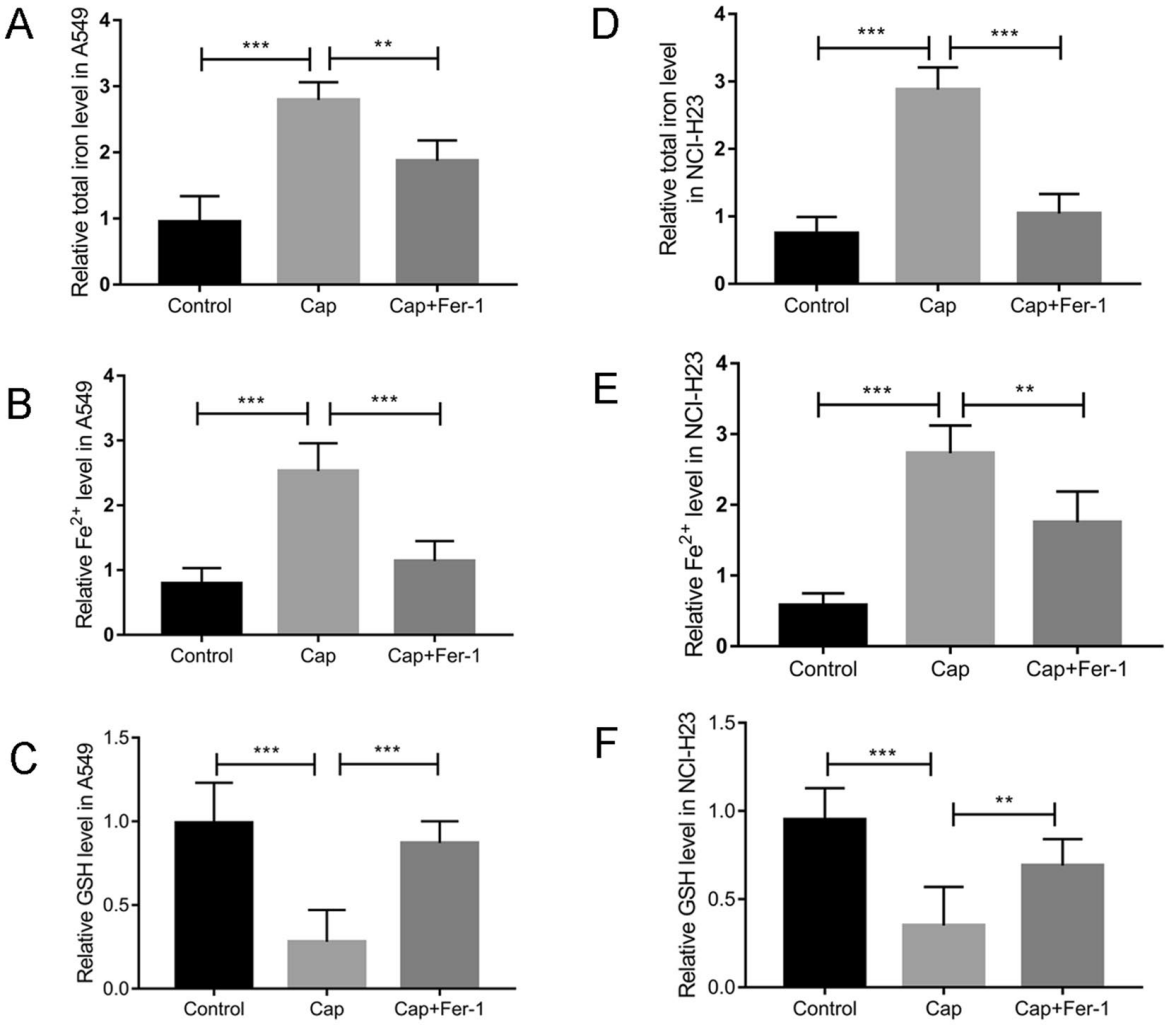
The changes of total iron levels, Fe^2+^ levels, and GSH levels in A549 and NCI-H23 cells. (**A** and **D**) The changes of total iron levels in A549 and NCI-H23 cells. (**B** and **E**) The changes of Fe^2+^ levels in A549 and NCI-H23 cells. (**C** and **F**) The changes of GSH levels in A549 and NCI-H23 cells. (**P < 0.01, ***P < 0.001).

According to the above results, capsaicin with concentration of 200 µM at 48 h was chosen for the subsequent studies in A549, while capsaicin with concentration of 100 µM at 48 h was chosen for the following researches in NCI-H23. After capsaicin treatment, the total iron levels in both A549 and NCI-H23 cells were significantly elevated in contrast with the control group (Fig. 2A,D), and the levels of ferric divalent ions were also significantly elevated (Fig. 2B,E). In the capsaicin + ferrostatin-1 group, the levels of total iron and divalent iron reduced significantly in contrast with those of capsaicin group (Fig. 2A,B,D,E). Subsequently, an ELISA kit was applied to survey the levels of GSH. In the capsaicin group, the levels of GSH were reduced significantly in both A549 and NCI-H23 cells. Such phenomenon was eliminated after ferrostatin-1 combined treatment in both A549 and NCI-H23 cells (Fig. 2C,F).

### Capsaicin affected the mRNA and protein levels of SLC7A11 and GPX4

**Figure 3:**
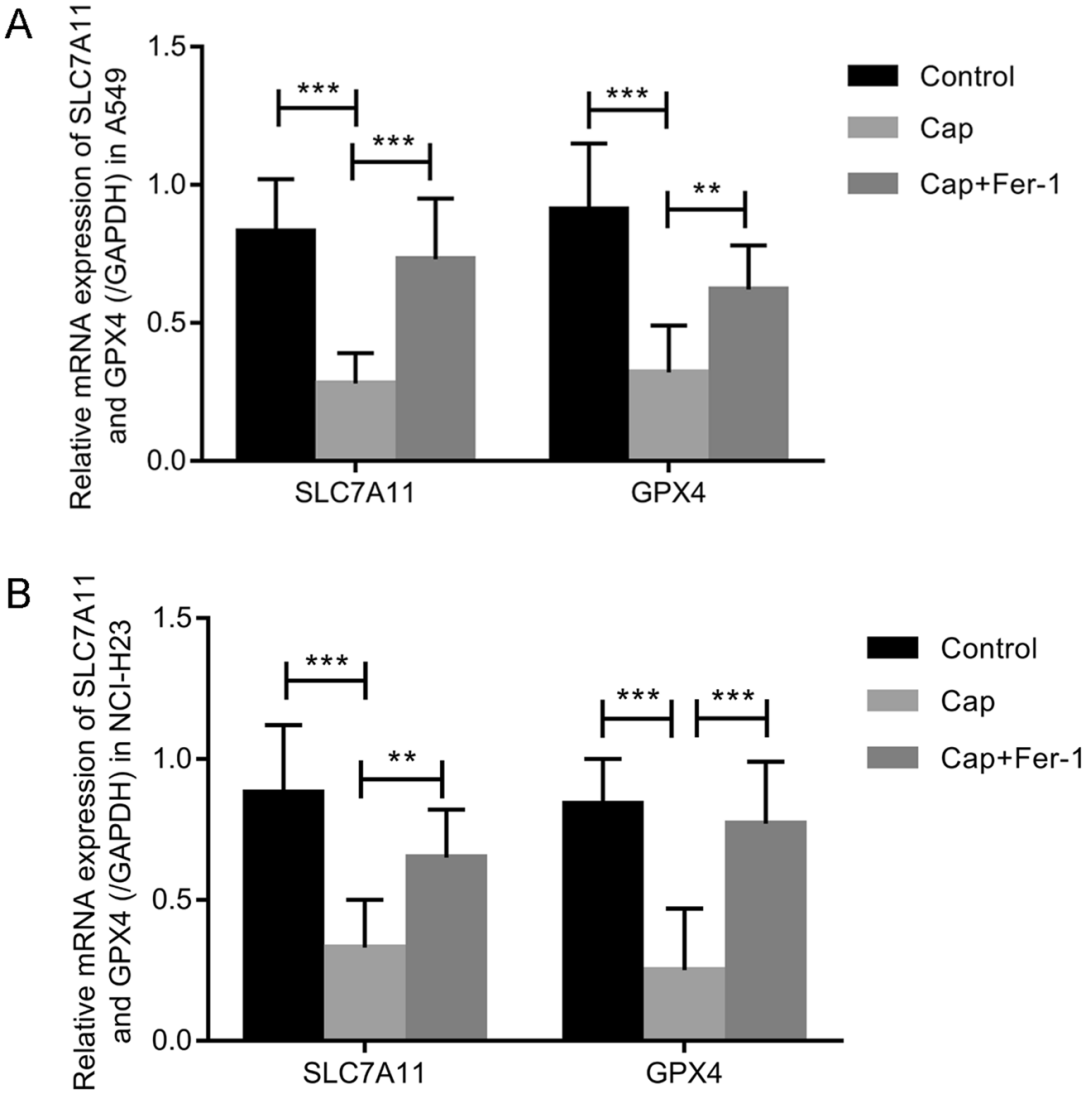
The expressions of SLC7A11 and GPX4 were analyzed by real-time PCR. (**A**) SLC7A11 and GPX4 mRNA expressions in A549 cells. (**B**) SLC7A11 and GPX4 mRNA expressions in NCI-H23 cells. (**P < 0.001, ***P < 0.0001).

**Figure 4:**
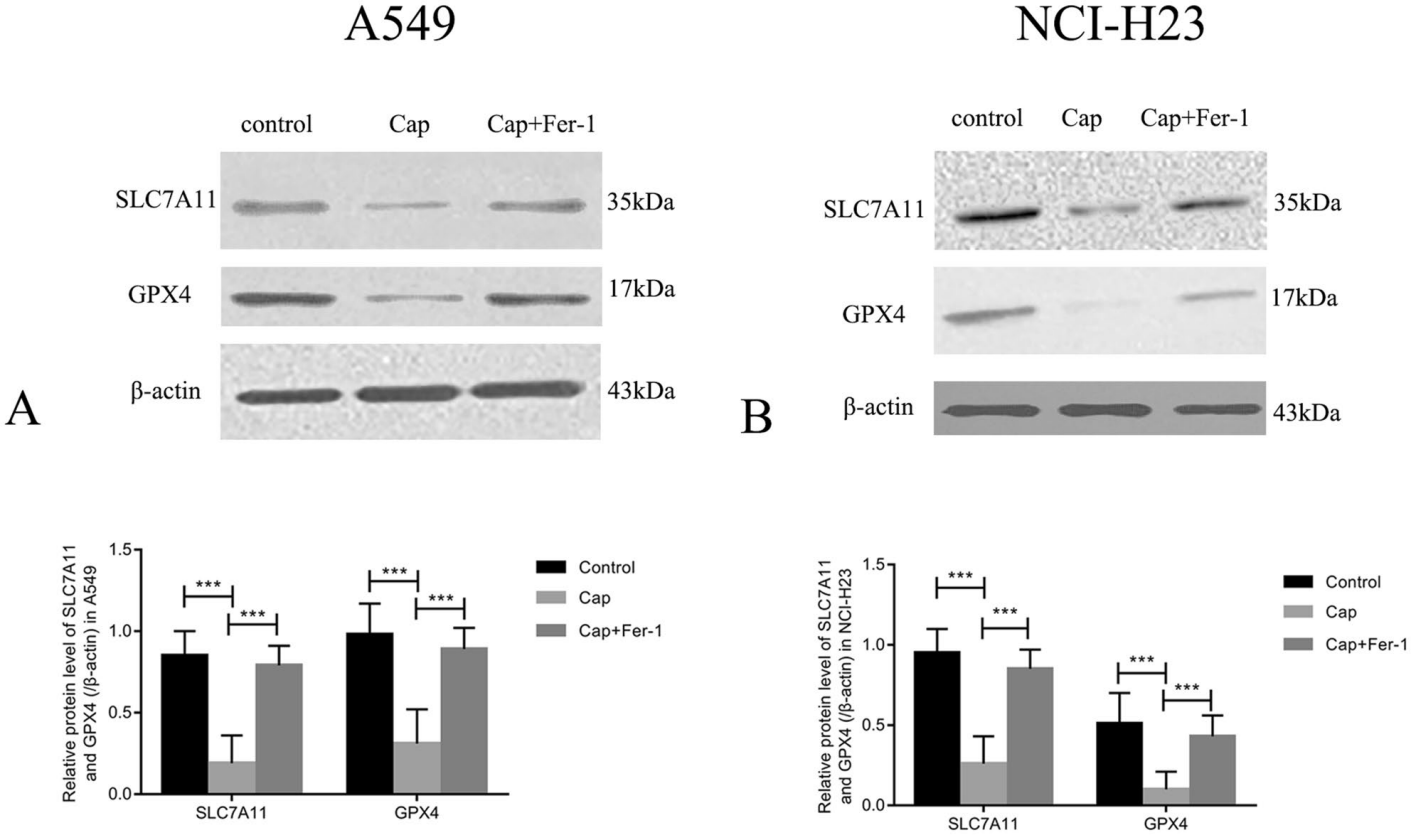
The expressions of SLC7A11 and GPX4 were analyzed by Western blotting analysis. (**A**) SLC7A11 and GPX4 protein expressions in A549 cells. (**B**) SLC7A11 and GPX4 protein expressions in NCI-H23 cells. (***P < 0.0001).

To find the molecular mechanisms of capsaicin-induced ferroptosis, we selected two critical signals from ferroptosis pathway, SLC7A11 and GPX4, as our research objectives. In this research, we performed real-time PCR to detect the mRNA levels of SLC7A11 and GPX4 in A549 and NCI-H23 cells treated with capsaicin. As demonstrated in Fig. 3A, SLC7A11 and GPX4 mRNA levels decreased significantly in A549 cells after capsaicin treatment, whereas ferrostatin-1 reversed such effect. Similarly, capsaicin led to significant changes in SLC7A11 and GPX4 expression levels in NCI-H23 cells, whereas ferrostatin-1 reversed such effect (Fig. 3B). The protein levels of SLC7A11 and GPX4 in A549 were down-regulated significantly in the capsaicin group in contrast with the control group, while ferrostatin-1 eliminated such effect (Fig. 4A). After capsaicin treatment, the protein levels of SLC7A11 and GPX4 in NCI-H23 were decreased significantly in contrast with the control group, whereas ferrostatin-1 reversed such effect (Fig. 4B).

## Discussion

Lung cancer, as one of the malignant carcinomas, has the highest cancer-related incidence and mortality in China. The 5-year survival rate of lung cancer is only 18% with current treatment options^25^. NSCLC is the most common pathological kind of lung cancer, whereas only about 20% of NSCLC patients have the surgical opportunity. Except for surgical strategy, radiotherapy and chemotherapy could be selected to treat NSCLC patients, which have great adverse reactions. So it is urgent to find new cure strategies for NSCLC patients. Capsaicin, the main component of chili pepper, has the anti-proliferative effect for numerous tumor cell lines^26^. Here, we studied the role of different capsaicin concentrations on A549 and NCI-H23 (the NSCLC cell lines), and ascertained the IC50 cytotoxic concentrations of capsaicin respectively. Through MTT assay, we discovered that the IC50 values of capsaicin for A549 cells was 200 μM for 48 h, while it was 100 μM and 24 h for NCI-H23 cells. It was revealed by the histogram that capsaicin could induce a concentration-dependent and time-dependent reduction in cell proliferation of both A549 and NCI-H23 cells. Ferroptosis and apoptosis are vital forms of cell death and take part in the survival and progression of tumor cells^27^. Here, we distinguished ferroptosis or apoptosis of A549 and NCI-H23 cells induced by capsaicin. Ferrostatin-1 is one of the inhibitors for ferroptosis, depending on its inhibition of lipid peroxides^28^. Z-VAD-FMK is one of the inhibitors for apoptosis. After the use of ferrostatin-1 and Z-VAD-FMK, we confirmed further that the anti-proliferative role of capsaicin on A549 and NCI-H23 cells was mainly due to ferroptosis but not apoptosis.

Ferroptosis was firstly proposed by Dixon^29^. It is a kind of iron-dependent programmed cell death, which is unlike apoptosis, necrosis and autophagy. Although iron is necessary for cell survival and cell viability, the accumulation of iron is one of the critical signs for ferroptosis. Ferroptosis is relative to the total iron level and iron divalent level, and excess iron accelerates the generation of superoxide and induces lipid peroxidation through free radicals in fenton reactive^30^. In our research, we found that there are significantly increased contents of total iron and iron divalent in both A549 and NCI-H23 cells after capsaicin treatment. These results verified again that ferroptosis is the cell death pattern of A549 and NCI-H23 cells induced by capsaicin. Such ferroptosis-dependent anti-cancer effect by capsaicin was also reported in U87-MG and U251 glioblastoma cells recently^31^, which came to the similar conclusion with us. Hence, capsaicin could exert its anti-cancer effect on different tumors in a ferroptosis-dependent way. Our discoveries here provided a theory basis for the therapeutic potential of capsaicin on a variety of tumors and add new science to existing literature. To further probe the molecular mechanism of the anti-proliferative effect of capsaicin on A549 and NCI-H23 cells, we observed the changes of SLC7A11 and GPX4, the key proteins involved in ferroptosis process.

SLC7A11 promotes the synthesis of GSH, counteracts the oxidative stress state in cells, and inhibits ferroptosis in cancer cells. SLC7A11 was overexpressed or up-regulated in patients suffered from NSCLC^32^, and silencing of SLC7A11 increased the curative effect of cisplatin to cancer cells^33^. Wang et al.^34^ found that inhibition of SLC7A11 triggers ferroptosis of cancer cells. Glutathione peroxidase (GPX4) is a kind of peroxidase decomposition enzyme widely existing in the body, and it prevents the excessive accumulation of intracellular lipid peroxides and finally inhibits ferroptosis^35^. Previous studies have found that chemotherapy resistance in malignant tumors is highly dependent on GPX4, whereas inactivation of GPX4 could eliminate cancer cells and prevent tumor recurrence in vivo^36^. According to the results of real-time PCR and western blot analysis, we found that SLC7A11 and GPX4 levels decreased significantly in A549 and NCI-H23 cells after capsaicin treatment in contrast with the control group. Hence, we concluded that capsaicin-mediated SLC7A11 and GPX4 down-regulation plays a critical role in regulating ferroptosis of A549 and NCI-H23 cells. Since GSH could regulate ferroptosis in cells by stimulate GPX4, consumption of intracellular GSH takes effect in activating ferroptosis^37^. We detected the levels of GSH and found that GSH levels decreased significantly in A549 and NCI-H23 cells after capsaicin treatment. It could be inferred that capsaicin induced A549 and NCI-H23 cells ferroptosis by regulating SLC7A11/GPX4 axis.

To sum up, these investigations verified that capsaicin has the anti-proliferative role on A549 and NCI-H23 cells by regulating SLC7A11/GPX4 signaling and finally leading to ferroptosis. In spite of these important findings, one of the main limitations of this research is that the effect of capsaicin in NSCLC animal model was not surveyed, so the future researches in vivo are necessary to put into effect. Our findings demonstrated that capsaicin might be used as a potential anticancer agent with ferroptosis-induced anti-proliferative effects in the treatment of NSCLC, so as to supply a novel cure strategy for NSCLC patients.

## Supplementary Information

Supplementary Information.

## Data Availability

The datasets generated and/or analyzed during the present study are available from the corresponding author upon reasonable request.
