# Supplementary material for: Capsaicin induces ferroptosis of NSCLC by regulating SLC7A11/GPX4 signaling in vitro
Source: Sci Rep. 2022 Jul 14;12:11996. doi: 10.1038/s41598-022-16372-3 (PMC9283462; doi:10.1038/s41598-022-16372-3)
Supplement: Supplementary file 1 — Supplementary Information. [file 41598_2022_16372_MOESM1_ESM.pdf]

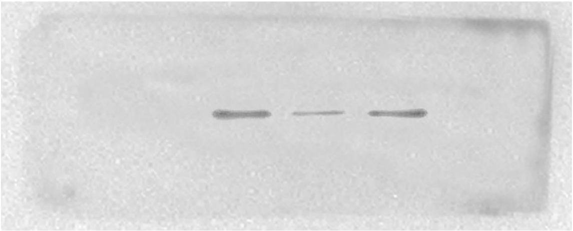

SLC7A11(A549)

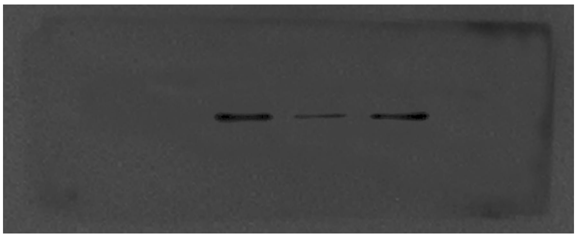

SLC7A11(A549) exposure 1

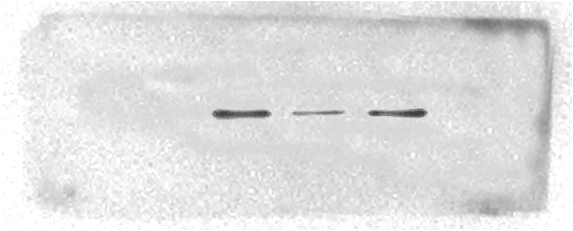

SLC7A11(A549) exposure 2

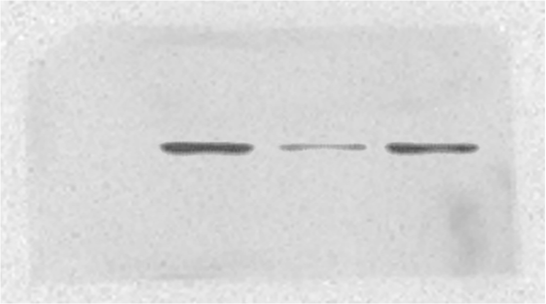

GPX4(A549)

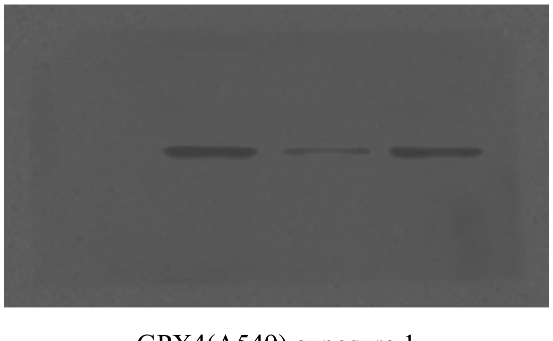

GPX4(A549) exposure 1

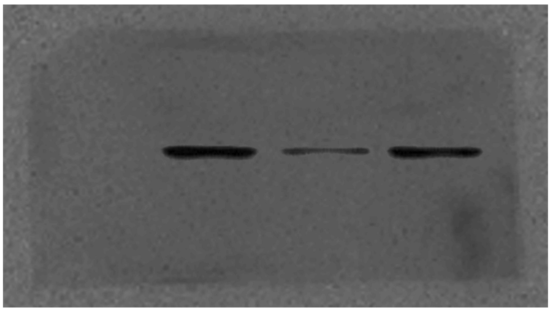

GPX4(A549) exposure 2

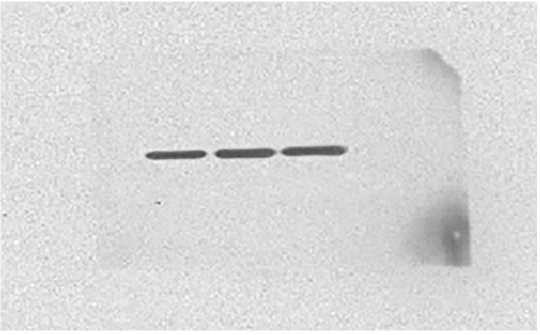

β-actin(A549)

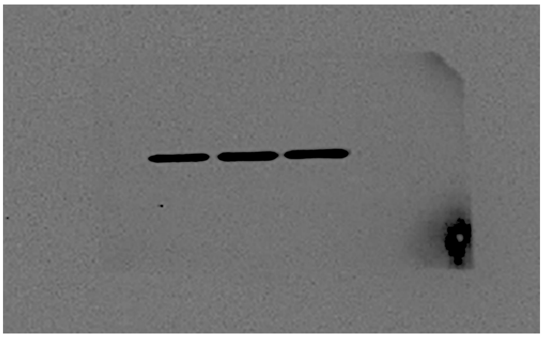

β-actin(A549) exposure 1

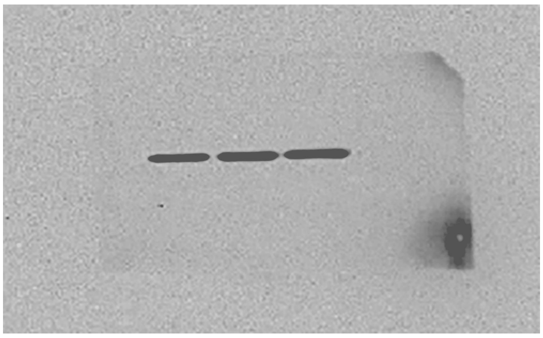

β-actin(A549) exposure 2

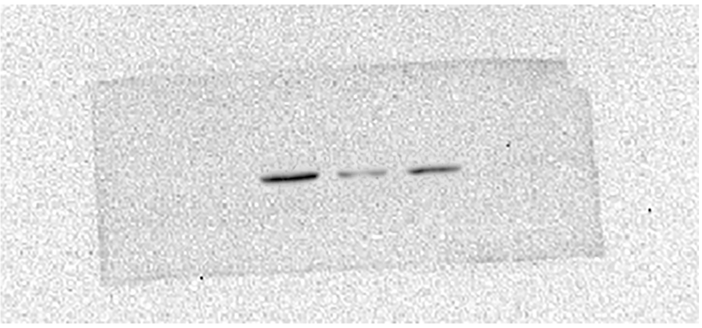

SLC7A11(NCI-H23)

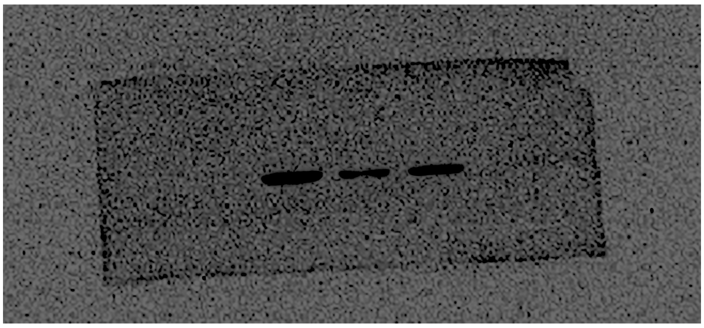

SLC7A11(NCI-H23) exposure 1

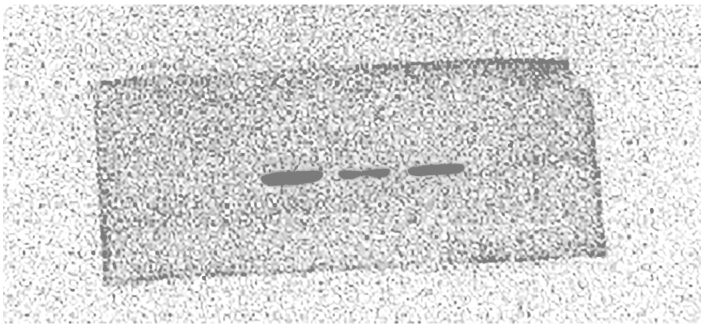

SLC7A11(NCI-H23) exposure 2

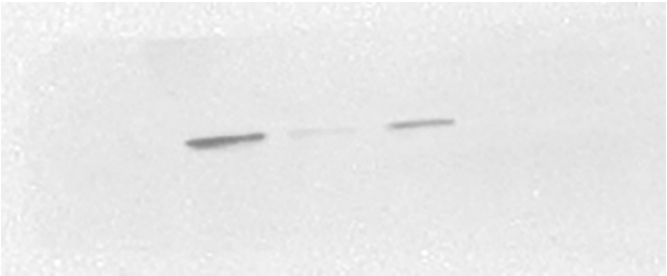

GPX4(NCI-H23)

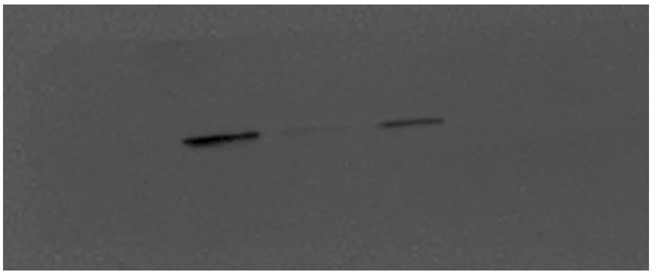

GPX4(NCI-H23) exposure 1

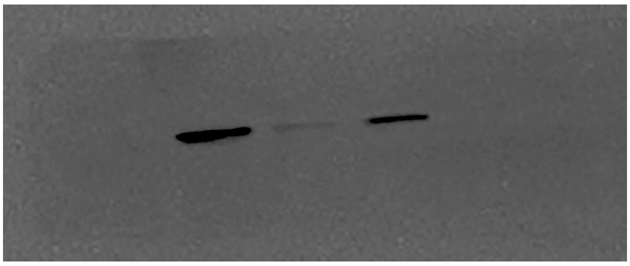

GPX4(NCI-H23) exposure 2

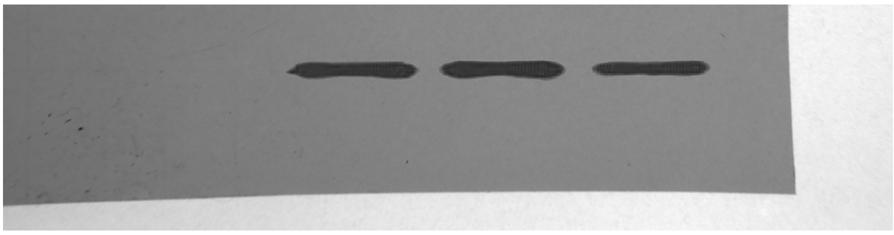

β-actin(NCI-H23)

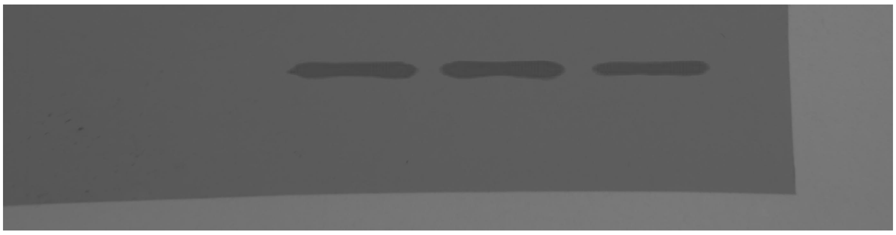

β-actin(NCI-H23) exposure 1

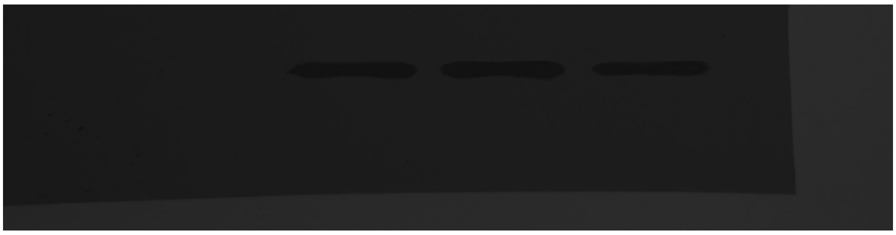

β-actin(NCI-H23) exposure 2
